# A permissive chromatin structure is adopted prior to site-specific DNA demethylation of developmentally expressed genes involved in macronuclear differentiation

**DOI:** 10.1186/1756-8935-6-5

**Published:** 2013-03-05

**Authors:** Aneta Bulic, Jan Postberg, Andreas Fischer, Franziska Jönsson, Günter Reuter, Hans J Lipps

**Affiliations:** 1 Institute of Cell Biology, Centre for Biomedical Education and Research, Witten/Herdecke University, Witten, Germany; 2 Helios Medical Centre Wuppertal, Paediatrics Centre, Witten/Herdecke University, Wuppertal, Germany; 3 Institute of Genetics, University Halle, Halle, Germany

**Keywords:** Cytosine methylation, Chromatin structure, Demethylation, Ciliates, Macronucleus

## Abstract

**Background:**

DNA methylation and demethylation are important epigenetic regulatory mechanisms in eukaryotic cells and, so far, only partially understood. We exploit the minimalistic biological ciliate system to understand the crosstalk between DNA modification and chromatin structure. In the macronucleus of these cells, the DNA is fragmented into individual short DNA molecules, each representing a functional expression and replication unit. Therefore, long range epigenomic interaction can be excluded in this system.

**Results:**

In the stichotrichous ciliate *Stylonychia lemnae*, cytosine methylation occurs in a small subset of macronuclear nanochromosomes expressed only during sexual reproduction. Methylation pattern shows similarity to that observed in fungi and *Drosophila*. Cytosine methylation correlates with gene activity and chromatin structure. Upon gene activation, cytosines become demethylated and a redistribution of histone post-translational modifications (PTMs) takes place. Evidence is presented that the formation of a permissive chromatin structure in the vicinity of the 5meCs precedes cytosine methylation and is probably a necessary prerequisite for their demethylation. Shortly after demethylation of cytosines occurs, the parental macronucleus degenerates, a new macronucleus is formed from a micronuclear derivative and the specific methylation pattern is transmitted from the germline micronucleus to the new macronucleus.

**Conclusions:**

We show that very few, or even only one, discrete methylated cytosines are required to assign regulatory functions at a specific locus. Furthermore, evidence is provided that a permissive chromatin structure is probably a necessary prerequisite for the demethylation of specific cytosines. Our results allow us to propose a mechanistic model for the biological function of cytosine methylation in the ciliate cell and its regulation during the cell cycle.

## Background

There is general agreement that differential molecular signatures of both the DNA and the proteinous contents of chromatin, such as histones, above the primary DNA sequence encode epigenetic information that are prerequisites for the spatiotemporal control of gene expression in a potentially heritable way. On the molecular level these signatures include DNA methylation at carbon number five in the pyrimidine ring of cytosines (5meC) [1], covalent post-translational modifications (PTMs) of all types of histone proteins, mostly at their N-terminal tails [2], and very probably the incorporation of specific histone variants into nucleosomal arrays [3]. DNA methylation has been frequently observed at symmetric CpG motifs in humans and many other organisms [4,5] as well as at asymmetric motifs (CpNpG and CpHpH), depending on the species and the developmental stage of an organism [6,7]. Cytosine methylation is mostly associated with transcriptional repression, possibly by direct blocking of transcription factor binding or by recruitment of histone deacetylases, thus impeding the decondensation of higher order conformations [1]. But it is now evident that cytosine methylation is more dynamic than previously thought [8].

The next level of epigenomic regulation of gene expression above the level of DNA modifications is the compaction of the 10 nm chromatin fiber, in which the DNA is wrapped around nucleosomes. This 10 nm fiber becomes further compacted by the interaction with linker histone H1 and other proteins [9]. Through altering the degree of chromatin compaction, PTMs of histone tails create chromatin structures favorable either for activation or repression of genes, depending on the genomic context and the combination of modifications at a given site [2]. Modifications found at distinct residues of the histone protein N-termini include - among others - lysine acetylation, lysine and arginine methylation as well as serine and threonine phosphorylation.

A crosstalk between DNA methylation and demethylation and chromatin structure has been assumed for a long time. In fact, histone modifications and DNA methylation could be mechanistically interconnected in various organisms. There are descriptions of DNA methylation directing histone modifications at specific loci in mammals [10-12] but only a limited number of examples have been reported in which histone modifications direct DNA methylation [13-17]. Demethylation processes are even more intricate as only one recent report shows that in *Arabidopsis,* a histone acetyltransferase regulates active DNA demethylation [18].

**Figure 1:**
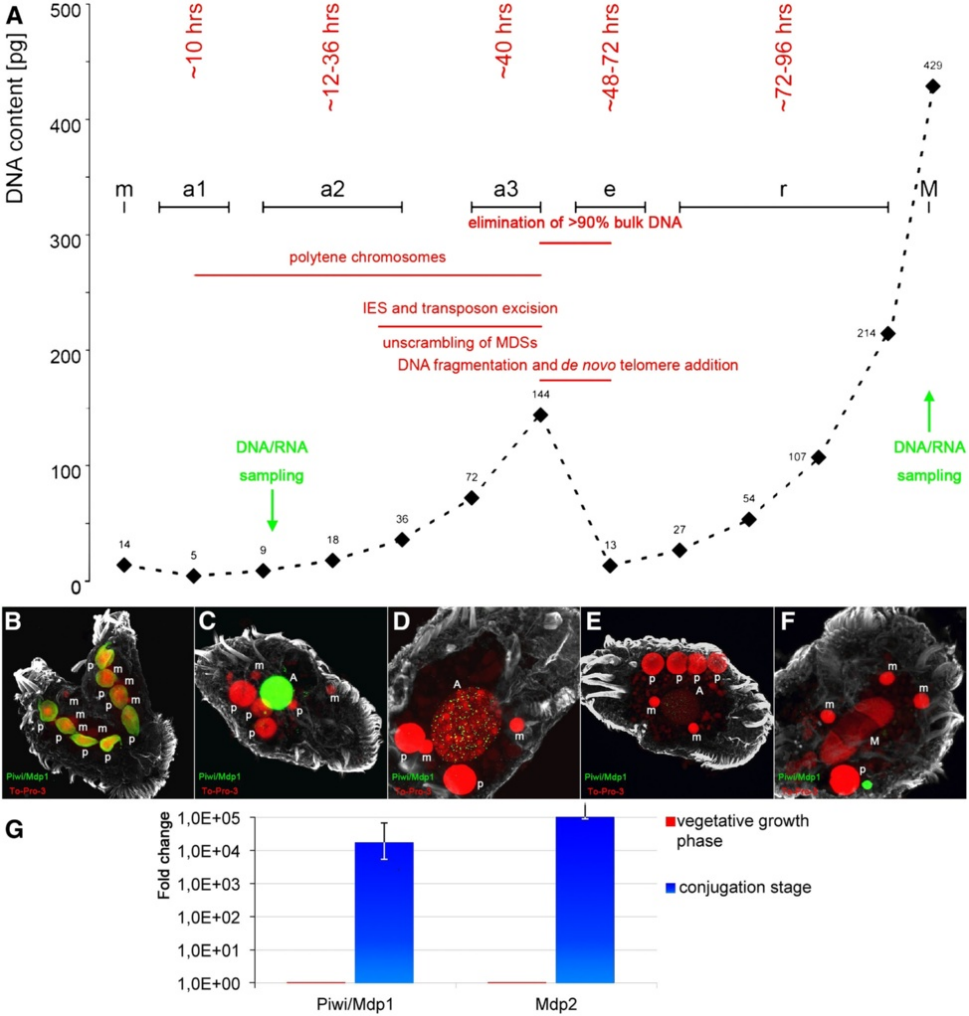
**Macronuclear differentiation in the stichotrichous ciliate *Stylonychia lemnae*.** (**A**) Schematic diagram of macronuclear differentiation. Time points at which DNA or RNA was isolated are indicated by an arrow. (**B**-**F**) Nuclear events during macronuclear differentiation and behavior of the developmentally expressed Mdp1 (Piwi) protein. (**B**) In the two conjugating cells, Piwi accumulates in the parental macronucleus (p). (**C**-**D**) During formation of polytene chromosomes, Piwi relocalizes from the parental macronucleus (p) to the developing macronucleus (a1-3). (**E**, **F**) During chromatin elimination in the anlage (e) and subsequent formation of the vegetative macronucleus (m), Piwi is no longer present. (**G**) Expression of the genes *mdp1* and *mdp2* during vegetative growth and at the onset of macronuclear differentiation. While no expression of these genes takes place during vegetative growth, they become transcribed approximately 30 hours after the onset of sexual reproduction. IES: sequences interrupting macronuclear-destined sequences in the micronuclear genome. MDS: macronuclear-destined sequences in the macronuclear genome.

To contribute to the debate on how the crosstalk between different levels of the epigenomic signature is mechanistically involved in the regulation of differential gene expression, we decided to exploit the minimalistic nature of ciliate macronuclear nanochromosomes, each representing an independent functional genetic unit [19]. Therefore, long-range epigenomic interactions can be excluded in this system, allowing us to directly analyze the relation between the different epigenetic modifications. Each ciliate cell contains two different types of nuclei, somatic macronuclei and germline micronuclei. All transcripts required for vegetative growth are derived from macronuclear DNA while the transcriptionally inert micronuclear DNA is organized into heterochromatin [20]. During sexual reproduction (conjugation) a new macronucleus (macronuclear anlage) differentiates from a micronuclear derivative while the old macronucleus becomes degraded in exconjugant cells after sexual reproduction. During macronuclear differentiation, dramatic DNA reorganization and elimination processes occur (Figure 1) resulting in a new macronucleus in which DNA is organized in short molecules, the nanochromosomes, ranging in size from between 0.4 to over 20 kb in stichotrichous ciliates. Each of these nanochromosomes usually contains only one open reading frame and all sequences required for expression and replication [21]. Conjugation is accompanied by a short-termed boost of differential expression of numerous genes [22]. The differential expression of selected genes (*mdp1* and *mdp2*) at the onset of sexual reproduction, which are repressed during vegetative growth, is a prerequisite for the regulation of subsequent developmental genome reorganization processes. Differential patterns of post-transcriptional histone H3 modifications were recently investigated in some of these genes, and these analyses revealed that gene activation leads to a relocalization of specific PTMs. In the transcriptionally repressed state, accumulation of active markers such as (H3K14ac, H3K4me3, H3K4me1) has been observed at its 3′-end, whereas those active markers relocate to the 5′-end during the activation of the gene [23].

Although cytosine methylation in the ciliate genome has been described as being potentially involved in DNA processing during macronuclear differentiation [24,25], a potential role in the regulation of gene expression has so far not been demonstrated. To address this issue, we analyzed the occurrence of 5-methyl cytosine in either constitutively expressed macronuclear genes or genes only activated during sexual reproduction. We provide strong evidence for a pivotal role of the DNA methylation status of specific cytosines for the regulation of differential gene expression and propose that site-specific cytosine methylation may be involved in long-term silencing of a sub-fraction of developmentally regulated genes. This methylation pattern seems to be transmitted from the micronucleus to the macronucleus during macronuclear differentiation. Moreover, we demonstrate that the creation of a permissive chromatin structure above the methylated cytosines precedes cytosine demethylation and may be a necessary prerequisite for this process.

## Results

The majority of macronuclear nanochromosomes are continuously expressed during vegetative growth of the cell. However, recently we showed that at the onset of sexual reproduction, ten hours post-conjugation a sub-fraction of nanochromosomes becomes developmentally expressed during early macronuclear development (Figure 1A, stage a1) while they are silent in the vegetative macronucleus. According to a recent microarray analysis, less than 1% of the nanochromosomes (approximately 100 nanochromosomes, most of which are not yet characterized) present in the macronucleus are developmentally expressed [22], for example, nanochromosomes encoding some histone variants or proteins involved in programmed DNA elimination show developmentally regulated expression.

**Figure 2:**
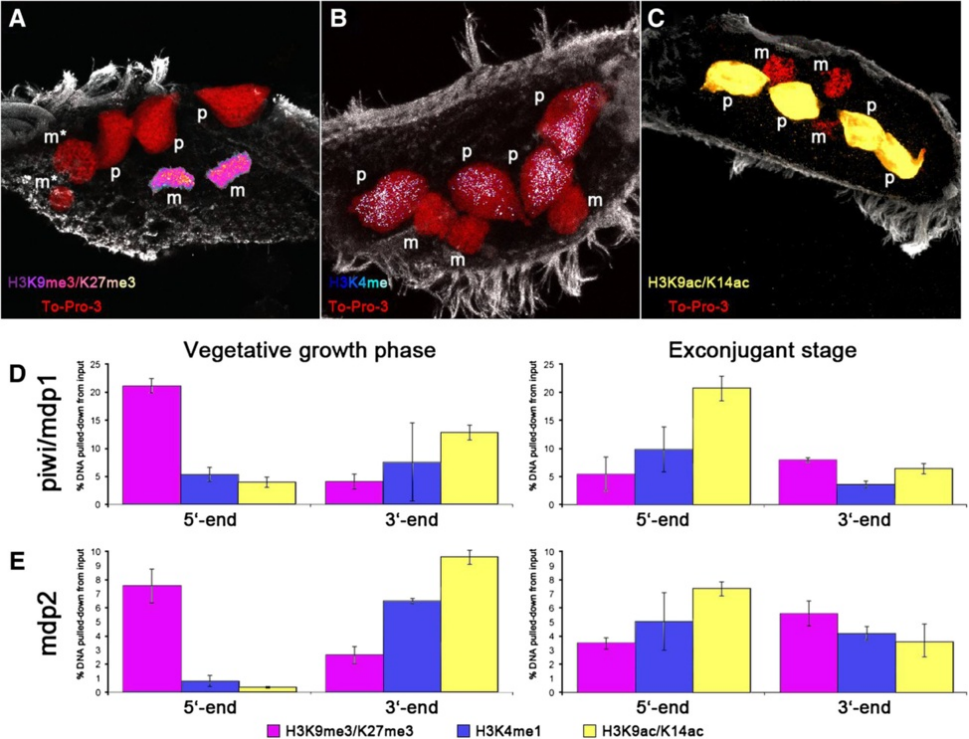
**Localization of different histone PTMs in parental macronuclei (p) and micronuclei (m) of exconjugant cells shortly after separation.** (**A**-**C**) *In situ* staining using antibodies directed against H3K9me3/K27me3 (**A**, hot magenta), H3K4me (**B**, hot blue), H3K9ac/K14ac (**C**, yellow). DNA counterstaining with To-Pro-3 (red). (**D**, **E**) Distribution of these PTMs on the 5^′^- and 3^′^-subtelomeric regions of *mdp1* as well as *mdp2* nanochromosomes during vegetative growth and in exconjugant cells.

When analyzing the distribution of PTMs typical for active chromatin on the well-characterized developmentally expressed nanochromosomes *mdp1* and *mdp2*, it became obvious that upon activation of gene expression, a redistribution of these PTMs takes place as has already been described [23]. While in the silenced state, these PTMs accumulate at the 3′-end, after activation they accumulate at the 5-′end. In addition, we now show that H3K9me3/K27me3, a PTM typical for repressed chromatin is found at the 5-′end in the silenced state but becomes removed upon activation (see below, Figure 2).

### In the macronucleus, 5meC is associated with some genes in their silent state and becomes removed upon activation

To find out whether there is also a possible link between cytosine methylation and gene expression, we tried to determine whether any cytosine methylation occurs in macronuclear DNA and whether this DNA modification differs between genes permanently expressed in the macronucleus from those which are transcriptionally repressed during vegetative growth and only activated during sexual reproduction.

**Figure 3:**
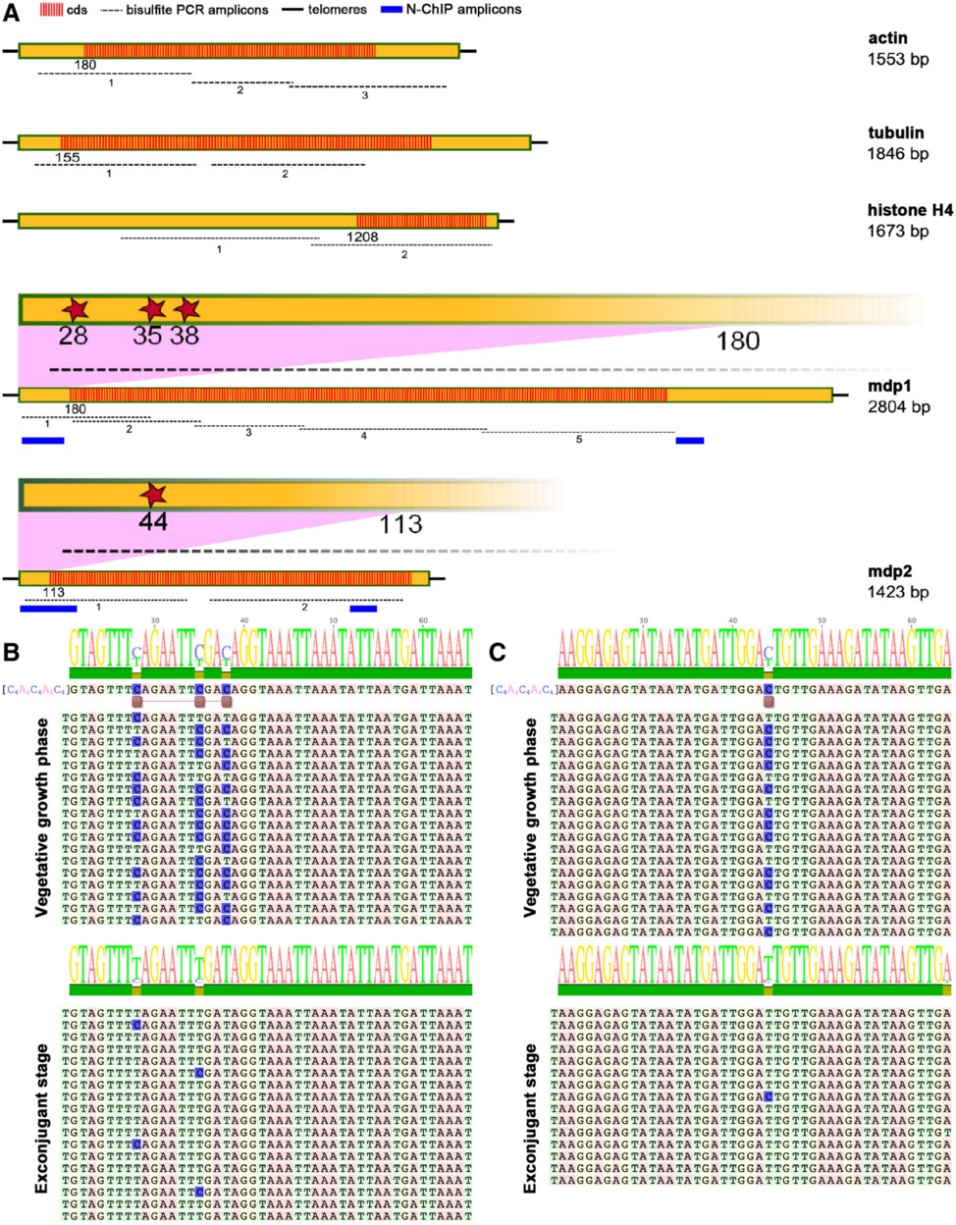
**Macronuclear nanochromosomes studied and DNA methylation pattern of developmentally expressed genes during vegetative growth and sexual reproduction.** (**A**) *Actin*, *tubulin* and *histone H4* are constitutively expressed genes; *mdp1* and *mdp2* are only expressed during sexual reproduction in exconjugant cells. Red shaded areas show the open reading frames of the nanochromosomes. Cytosines are methylated at positions 28, 35 and 38 in *mdp1* and at position 44 in *mdp2* (red stars). Black dashed lines: amplicons analyzed for cytosine methylation; blue lines: amplicons used for ChIP analyses. (**B**, **C**) DNA methylation pattern in *mdp1* (**B**) and *mdp2* (**C**) during vegetative growth (*mdp1/mdp2* repressed) and in exconjugant cells (*mdp1/mdp2* expressed).

For a long time it was believed that no cytosine methylation is present in the ciliate genome, and only recently has cytosine methylation in the micronucleus and the developing macronucleus been described as being associated with DNA sequences becoming eliminated during further development [24,25]. In RP-HPLC analyses of macronuclear DNA, no cytosine methylation could be detected [24] (data not shown) suggesting that it is present only in very low quantities (below 0.2%) and in very few genes in the macronucleus. The most likely methylated genes should be those silenced during vegetative growth in macronuclear DNA but activated only during sexual reproduction. We therefore decided to look for cytosine methylation in those genes silenced in the vegetative macronucleus and compare them to constitutively expressed genes. To establish the 5-methyl cytosine status, macronuclear DNA from vegetative cells and from cells during an early stage of macronuclear development (exconjugant cells) was subjected to bisulfite treatment. Five specific macronuclear nanochromosomes (Figure 3A) were amplified by PCR using primers specific for bisulfite-modified DNA and cloned. Between eight and eighteen clones were sequenced for each analysis. Three of the analyzed nanochromosomes encode genes constitutively expressed in the macronucleus, *actin I* [GenBank accession number DQ108616], *β tubulin* [GenBank accession number AF10208.1] and *histone H4* [GenBank accession number X16018], the other two, *mdp1* [GenBank accession number AY261996] and *mdp2* [GenBank accession number GU111958] belong to the most prominent genes expressed only during sexual reproduction. The expression of these two genes during vegetative growth and the onset of sexual reproduction is shown in (Figure 1G). Furthermore, the redistribution of PTMs typical for active chromatin was shown before for both nanochromosomes [23]. Mdp1 encodes a Piwi/argonaute family protein that is involved in a transnuclear crosstalk and the metabolism of small RNAs [26]. Its spatiotemporal localization is shown in Figure 1B-F. During conjugation Piwi/MDP1 accumulates in the parental macronucleus fragments (Figure 1B), and at later stages it relocalizes from the parental macronucleus to the new developing macronucleus (Figure 1C-E), but is no longer present during the second round of DNA amplification in the new macronucleus (Figure 1F). Thus, it behaves as expected from the scanRNA model for the control of macronuclear differentiation in which small RNAs associate with Piwi are involved in specifying the processing of the micronuclear genome during macronuclear development [21]. MDP2 is functionally less characterized, but homology searches suggest that it contains an Alba protein domain and therefore may be a cytoplasmic RNA binding protein [27]. Silencing expression of both of these genes during sexual reproduction leads to an arrest in macronuclear differentiation in the early polytene chromosome stage (Figure 1A, stage a2).

Analyses of bisulfite-modified macronuclear DNA revealed that in the genes constitutively expressed (*actin I, β tubulin, histone H4*) no cytosine methylation can be detected in either vegetative cells or in exconjugant cells. In contrast, in DNA isolated from vegetative cells, cytosine methylation was observed in the 5′-non coding region of both *mdp1* and *mdp2*. In *mdp1*, three methylated cytosines were found at positions 28, 35 and 38 in the sequence context CAG and CG. In *mdp2*, only one methylated cytosine could be found at position 44 in the sequence context CTG (for statistical significance see Additional file 1). In both cases the methylated cytosines are upstream of the putative TATA boxes. No sequence homology between *mdp1* and *mdp2* in the regions in which these methylated cytosines were located could be found except that both sequences are very AT rich. In both cases the first cytosine(s) downstream, the telomeric sequence was methylated while no cytosines further downstream were modified (Figure 3B, C). Remarkably, although the enormous multiplication of both *mdp1* and *mdp2* expression in exconjugant cells strongly indicate that both genes are silent during vegetative growth (Figure 1G), we could find a significant subset of *mdp2* nanochromosomes unmethylated (Figure 3C; Additional file 1), suggesting that at least for some genes, DNA methylation alone is not sufficient to induce repression of their expression.

In DNA isolated from exconjugants at a time point where *mdp1* and *mdp2* are expressed, no cytosine DNA methylation could be observed, suggesting that they are actively demethylated (Figure 3B, C). Thus, demethylation of very few, or in the case of *mdp2*, even only one methyl group at a specific cytosine correlates with activation of gene expression. Interestingly, it has been reported that induced CD4+ T-cell activation leads to demethylation of a single CpG site in the promoter-enhancer of the human *IL2* gene, and that this change is necessary and sufficient to enhance transcription of a reporter plasmid [28].

### DNA methylation signatures of *mdp1* and *mdp2* nanochromosomes are reminiscent of their micronuclear patterns

We made an attempt to understand how this specific methylation pattern is introduced into these developmentally expressed nanochromosomes. For this, we isolated DNA from micronuclei and early macronuclear anlagen in the precursor sequences of *mdp1* and *mdp2*. As shown in micronuclear DNA as well as in DNA from the differentiating macronucleus, the same cytosines are methylated as in the vegetative macronucleus. Our data therefore suggest that the methylation pattern of the germline micronucleus is preserved and transmitted to the new macronucleus. In contrast, in the nanochromosomal precursor sequences, macronuclear-destined sequences (MDSs) of either the micronucleus or the developing macronucleus of the constitutively expressed *β tubulin*, no methylation was found in either the micronuclei or macronuclear anlage (see Additional file 2).

### Repressive chromatin markers become relocalized in a subset of genes which are activated at the onset of sexual reproduction only

We have recently shown that activation of *mdp1* and *mdp2* correlates with a redistribution of histone modifications typical for active genes. While in the silenced status, these PTMs accumulate at the 3′-end of the gene they are enriched at the 5′-end upon activation [23]. In this former study the distribution of modifications typical for repressed chromatin was not included. In fact, no signals are obtained when macronuclei of vegetative cells (data not shown) or fragments of parental macronuclei are stained *in situ* with an antibody directed against H3K9me3/K27me3 (Figure 2A), suggesting that these PTM do not occur or only in minor concentrations in the macronucleus; a very similar situation to that of the methylated cytosines. Notably, unlike in many animals studied so far, a functional discrimination between H3K9me3 and H3K27me3 has not been demonstrated in ciliated protozoa to date [20,29]. Using ChIP experiments we now analyzed the distribution of these repressive markers in combination with PTMs typical for active chromatin (H3K4me in Figure 2B, H3K9ac/K14ac in Figure 2C) on the *mdp1* and *mdp2* nanochromosomes either in their silenced or active state (Figure 2D, E). In the silenced status we observe an enrichment of the active marker at the 3′- end similar to those observations reported for other PTMs typical for active chromatin [23], but interestingly, enrichment of H3K9me3/K27me3 could be detected on the 5′-end of both *mdp1* and *mdp2* nanochromosomes at a similar position to that where we also find methylated cytosines (Figure 2D and E, vegetative growth phase). Upon activation, the concentration of H3K9me3/K27me3 at the 5′-end is greatly reduced while H3K4me and H3K9ac/K14ac relocalize from the 3′- to the 5′-end. No H3K9me3/K27me3 associated with constitutively expressed genes could be found, while the distribution of active PTMs was similar to those expressed in *mdp1* and *mdp2* (data not shown) [23]. Thus we conclude that PTMs typical for repressive chromatin are involved in the regulation of a small sub-fraction of macronuclear genes which exhibit short-term expression during sexual reproduction but are repressed during most of the cell’s life cycle.

### Inhibition of HAT activity impedes DNA demethylation as well as activation of *mdp1* and *mdp2* expression

**Figure 4:**
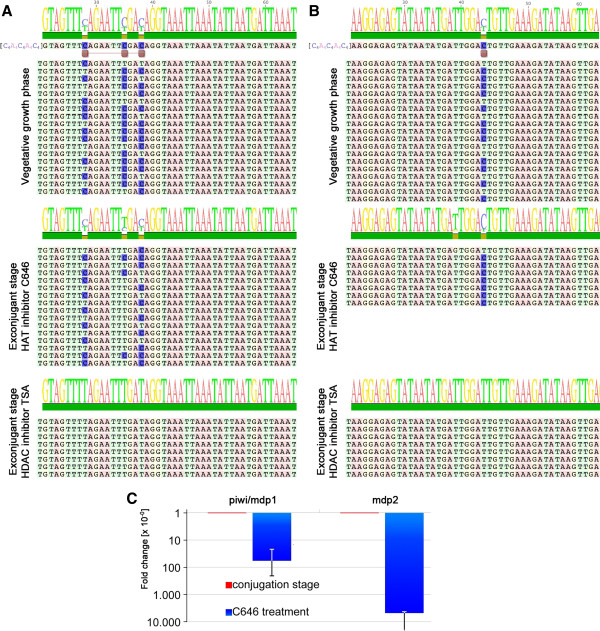
**Inhibition of histone acetyltransferase and histone deacetylase.** (**A**, **B**) Effect of histone acetyl transferase inhibitor C646 and histone deacetylase inhibitor TSA on the DNA methylation pattern of *mdp1* (**A**) and *mdp2* (**B**). (**C**) The effect of histone deacetylase inhibitor C646 on the expression of these nanochromosomes during sexual reproduction was analyzed by qPCR. In both cases a dramatic decline in expression is observed after treatment with C646.

It is generally assumed that there is a crosstalk between cytosine methylation and chromatin structure, determined by PTMs and other proteins. To elucidate this mechanism in our biological system, we used specific drugs which either inhibit histone acetyltransferase (HAT) or histone deacetylase (HDAC) activity. C646 is a p300 histone acetyltransferase inhibitor. Histone acetyltransferase (HAT) p300/CBP is a transcriptional co-activator implicated in many gene regulatory pathways with acetyltransferase activity. It was shown that C646 suppresses histone H3 and H4 acetylation in mouse fibroblast cell lines [30]. Trichostatin A (TSA) belongs to the class of histone deacetylase inhibitors that have broad activity spectra. TSA selectively inhibits members of the mammalian class I and II HDAC families, whereby it can alter gene expression by interfering with the removal of acetyl groups from histones, and thereby changes the accessibility of transcription factors to the DNA. At the concentrations used in this study, both inhibitors show no effect on vegetative cells but exhibit a phenotype in exconjugant cells. Macronuclear development is slightly delayed during the first DNA amplification stage and is arrested in the polytene chromosome stage of the macronuclear anlage (Figure 1A). Upon TSA treatment, IES (internal eliminated sequence) excision is defective [31] and incubation of cells with the C646 inhibitor at the beginning of sexual reproduction largely prevents the accumulation of H3K9ac/K14ac at the 5′-end of this stage (Additional file 3). Furthermore, we observed that methylated cytosines at both nanochromosomes *mdp1* and *mdp2* remained present during macronuclear development, whereas after incubation with TSA the DNA remained unmethylated (Figure 4A, B). We also investigated the effect of C646 on the expression of *mdp1* and *mdp2*. For normalization, we included expression analyses of the two housekeeping genes *actin I* and *β tubulin*. As shown in Figure 4C, we observed very low level *mdp1* and *mdp2* expression when compared with untreated cells at the conjugation stage. Hence, we concluded that both genes remained silenced upon inhibition of acetyltransferase, pointing to a crosstalk between PTMs and methylation status.

## Discussion

Until very recently it was believed that cytosine methylation does not occur in ciliated protozoa, or only at a defined stage of development [32]. Here we show that this DNA modification occurs also in a small subset of macronuclear nanochromosomes developmentally expressed during sexual reproduction (Figure 3), while no such DNA modification is observed in constitutively expressed nanochromosomes. Therefore, as in other organisms, cytosine methylation also correlates with transcriptional activity in the ciliate cell. Surprisingly, only very few, or in the case of *mdp2*, only one methylated cytosine are found in the 5′-subtelomeric regions of the nanochromosomes upstream the TATA box, and removal of these singular methyl groups correlates with activation of expression. This indicates that only very few and discrete methylated cytosines are required to assign regulatory functions to a specific locus. Such a low level of methylated cytosines is below the detection limit of the standard RP-HPLC used in this study which could explain the failure of cytosine methylation detection in vegetative cells in former studies [24]. While in mammals methylated cytosines are preferentially found in the CpG context, we find them also in the CAG or CTG context, somehow similar to what has been described in *Neurospora*, plants or *Drosophila*[8,33,34]. The presence of methylated cytosines correlates with the distribution of histone PTMs on the nanochromosomes. While in nanochromosomes active throughout the cell’s life PTMs typical for active chromatin accumulate at the 5′-end and no PTMs typical for repressed chromatin are found, active PTMs accumulate at the 3′-end of the nanochromosomes in the presence of 5meC and a PTM typical for repressed chromatin is present at the 5′-end above the methylated cytosines (Figure 2). Upon activation of expression, not only cytosines become demethylated but PTMs also redistribute on the nanochromosome. H3K9me3/K27me3, typical for repressed chromatin becomes removed and the active PTMs, H3K4me and H3K9ac/K14ac, are removed from the 3′-end and accumulate at the 5′-end or at least are equally distributed along the entire nanochromosome as in the case of *mdp2* (Figure 2) [23]. The combined presence of 5meC as well as H3K9me3/H3K27me3 at the 5′-end of nanochromosomes could be relevant regarding the effectiveness of gene silencing. This could explain our observation that a significant subset of *mdp2* nanochromosomes without 5meC was present in vegetative cells, whereby no expression of this gene above threshold could be detected. To understand the relationship between cytosine demethylation and chromatin context, we either inhibited histone acetylation or histone deacetylation. Until very recently, such a relationship remained unclear but now it has been shown in *Arabidopsis* that H3 acetylation creates a chromatin environment permissible for 5-methyl cytosine DNA glycolysis [18]. Our results suggest a similar mechanism, that is, after inhibition of acetyl transferase, which prevents the accumulation and redistribution of H3K9ac/14ac at the 5′-end, thereby creating a permissive chromatin structure, demethylation cannot be observed. But these observations do not completely exclude the possibility that the drugs applied may affect other pathways involved in DNA methylation or demethylation. A relevant question is how a specific methylation pattern is established in a small subset of nanochromosomes. We show that a similar methylation pattern has already been observed in the macronuclear precursor sequences in the micronucleus and the developing macronucleus. Shortly after activation of the genes developmentally expressed during sexual reproduction, the old macronucleus degenerates and a new macronucleus is formed by a micronuclear derivative and it seems reasonable to assume that the specific methylation pattern is transmitted from the germline micronucleus through macronuclear development towards the mature, vegetative macronucleus. As such, no specific *de novo* methylation has to occur. Taking all presented results together allows us to propose a mechanistic model for the biological function of cytosine methylation in the ciliate cell and its regulation throughout the life cycle of these single cell eukaryotes. The presence of the methylated cytosines correlates with gene activity and suggests that very few, or even a single methylated cytosine mark, is sufficient for long-term repression of gene expression, thus adding one further example of a specific cytosine correlating with gene expression [28]. Upon gene activation, a relocalization of histone PTMs takes place and assembly of a permissive chromatin structure above the methylated cytosines, a necessary prerequisite for subsequent demethylation takes place. This mechanism has also recently been suggested for *Arabidopsis*. Shortly after this demethylation event, the old macronucleus disintegrates and the specific methylation pattern is transmitted from the micronucleus to the vegetative macronucleus during nuclear differentiation.

## Conclusions

We introduce a biological model system, ciliated protozoa, whose minimalistic nature of macronuclear genome organization seems to be especially suited to analyze the crosstalk between different levels of epigenomic regulation. Macronucleus DNA is fragmented into small nanochromosomes, each representing a functional, transcriptional and replicational unit. We show that a low level of cytosine methylation occurs in the subtelomeric region of a small subset of macronuclear nanochromosomes only expressed during sexual reproduction, and this specific methylation pattern seems to be passed on from the germline nucleus to the vegetative macronucleus. Upon gene activation these cytosines become demethylated, demonstrating that only very few and discrete methylated cytosines are required to assign regulatory functions at a given locus. Cytosine methylation and demethylation also correlate with chromatin structure. Upon gene activation, a permissive chromatin structure is formed prior to cytosine demethylation and may be a prerequisite for this process. Results obtained in this study may not only be relevant for ciliated protozoa but for eukaryotes in general.

## Methods

### Growth of *Stylonychia*, synchronization, administration of drugs

Growth of *Stylonychia lemnae* and isolation of macronuclei, micronuclei or macronuclear anlagen were performed as described previously [32]. DNA contamination of other nuclear types was avoided by purification of macronuclear, micronuclear or macronuclear anlagen DNA by electroelution from agarose gels. Purification was always repeated twice. To set up conjugation, cells of different mating types were mixed. Conjugation efficiency was between 90 and 95%. Cells from various stages of macronuclear development as well as vegetative cells were used to isolate total RNA, DNA and chromatin.

### Inhibition of histone deacetylase and histone acetyl transferase

In some experiments, ciliates were treated with histone deacetylase (HDAC) inhibitor trichostatin A (TSA) (final concentration 0.4 μM, administered during conjugation), or histone acetyl transferase (HAT) inhibitor C646 (Calbiochem, Merck Millipore, Darmstadt, Germany) (final concentration 3 μM, added two hours prior to conjugation set up). RNA and DNA from such treated cells were isolated 30 hours post conjugation.

### Purification of nucleic acids and cDNA synthesis

Isolation of DNA was performed as described [24]. RNA was isolated from exconjugant cells harvested 30 hours post conjugation. Total RNA was isolated using InnuSOLV RNA reagent (Analytik Jena, Jena, Germany) according to the manufacturer’s recommendations. From obtained RNA, genomic DNA was removed using RNase-free DNase I (Fermentas, Thermo Fisher Scientific, Waltham, MA, USA). 2 μg of RNA were used to synthesize cDNA with InnuSCRIPT reverse transcriptase (Analytik Jena, Jena, Germany).

### Analyses of gene expression

200 ng of cDNA were used for each quantitative real-time PCR (qPCR) reaction using a Light Cycler instrument (Roche, Mannheim, Germany) as described previously [23]. To generate standard curves, undiluted input DNA of known concentration or serial dilutions (1/10; 1/100) were analyzed at least in triplicates. *Actin I* and *α-tubulin* were used as housekeeping genes in order to determine the relative amount of expression of genes of interest. We performed qPCR using the following primers (see supplementary material Table 1): Initial denaturation was carried out for 5 minutes at 95°C and subsequently 45 cycles were performed (95°C for 10 seconds; 60°C for 15 seconds), then a final elongation at 72°C for 30 seconds was allowed.

**Table 1:** Macronucleus specific primers for the N-ChIP analyses

**Position**	**Primer**	**Sequence**
mdp1 5′	Piwi a	5′-CCGTAGTTTCAGAATTCGACAGG-3′
mdp1 5′	Piwi b	5′-GTTGAGGCCTCGACAACTTAAAA-3′
mdp1 3′	Piwi3′-UTR	5′-GTAGGGTCTCTCATCTCCTGTTCGC-3′
mdp1 3′	Piwif	5′-CTTGTCTGGTGTATCACCGATACCATC-3′
mdp2 5′	mdp2-1a	5′- CTTGTCTGGTGTATCACCGATACCATC- 3′
mdp2 5′	mdp2-b	5′-TGCTTGACTGAGTCGTCAGAAT-3′
mdp2 3′	mdp2-k	5′-AGAAGAGGAGGACCGAGTGG-3′
mdp2 3′	mdp2-l	5′-ATCAGTCTCTGAGGGAAATAGGC-3′

### Bisulfite sequencing

Bisulfite treatment was performed with 1 μg macronuclear DNA using EpiTect (Qiagen, Hilden, Germany) according to manufacturer’s recommendations. Bisulfite converted DNA was amplified using conversion specific primers (see supplementary material Table 2) designed with the Kismeth software (http://katahdin.mssm.edu/kismeth), considering potential cytosine methylation in any sequence context (CG, CHG, and CHH). (For bisulfite primer amplicons, see Figure 3). Amplicons were sequenced (Eurofins MWG Operon, Ebersberg, Germany), constructs were aligned using Geneious v5.6 software (Geneious, Auckland, New Zealand) and the Clustal W algorithm (See Figures 3 and 4 for aligned sequences).

**Table 2:** Macronucleus specific primers for bisulfite analysis

**Position**	**Primer**	**Sequence**
1	Actin 1 for	5′-GAGAGTATTAGATGTATTGATTAGG-3′
1	Actin 1 rev	5′-AATTTAAATCATCTTCTCTCTATTAATC-3′
2	Actin 2 for	5′-GATTAATAGAGAGAAGATGATTTAAATT-3′
2	Actin 2 rev	5′-AAAATCTCTTCTAACATCAACATC-3
3	Actin 3 for	5′-GATGTTGATGTTAGAAGAGATTTT-3′
3	Actin 3 rev	5′-CCCCAAAACCCCATTTAATAA-3′
1	Tubulin 1 for	5′-TTTGAATATAAGGATATTAATTTTAAGAA-3′
1	Tubulin 1 rev	5′-ATTCCCATACCTAAACCAATAC-3′
2	Tubulin 2 for	5′-GTATTGGTTTAGGTATGGGAAT-3′
2	Tubulin 2 rev	5′-ATAAATTATTCACCAACTCTCTTA-3′
1	Histone 1 for	5′-GTTTAAAGGATTTGAAAGGAAAAAAGATT-3′
1	Histone 1 rev	5′-CCCTAAAAACTTCATAATATCTCC-3′
2	Histone 2 for	5′-GGAGATATTATGAAGTTTTTAGGG-3′
2	Histone 2 rev	5′-AACATATCAAAAACTTCATCCTC-3′
1	Mdp1 1 for	5′-TCCAACTTCTCCTTTATCTTCTCTTTCACA-3′
1	Mdp1 1 rev	5′-GTAGTTTYAGAATTYGAYAGGTAAATTAAATA-3′
2	Mdp1 2 for	5′-GGGATTAGTATTAATAGTGTTTTTTAAG-3′
2	Mdp1 2 rev	5′-AAATCTCAATCCTCTCATAAATAAATTA-3′
3	Mdp1 3 for	5′-TAATTTATTTATGAGAGGATTGAGATTT-3′
3	Mdp1 3 rev	5′-AAACTCATTAAAAAATCAACTCTCTC-3′
4	Mdp1 4 for	5′-GAGAGAGTTGATTTTTTAATGAGTTT-3′
4	Mdp1 4 rev	5′-ATCATAAATAAAATCTTCCCTTTC-3′
5	Mdp1 5 for	5′-GAAAGGGAAGATTTTATTTTATGAT-3′
5	Mdp1 5 rev	5′-GAGATGTGGGATCTTTCAAAAACCC-3′
1	Mdp2 1 for	5′-AAGGAGAGTATAATATGATTGGAYTGTTGA-3′
1	Mdp2 1 rev	5′-AATCAATATTCCTAACATATCTTCTC-3′
2	Mdp2 2 for	5′-GAGAAGATATGTTAGGAATATTGATT-3′
2	Mdp2 2 rev	5′-ATCCTCCTCTTCTCATACC-3′

### Chromatin isolation and ChIP

Chromatin isolation and ChIP analyses were carried out as described [23]. Antibodies used for ChIP were directed against H3K9me3/K27me3, H3K4me or H3K9ac/K14ac (Abcam, Cambridge, UK). The specificities of these polyclonal antibodies on ciliate histone modifications are well documented [20].

### Confocal laser scanning microscopy

Sample treatment for immunofluorescence and subsequent analyses by confocal laser scanning microscopy (CLSM) was performed using a protocol, antibodies and dyes, which are described in detail in [20]. Images were assembled using ImageJ (Rasband, WS, ImageJ, National Institutes of Health, Bethesda, MD, USA, http://rsb.info.nih.gov/ij/, 1997–2004) and Adobe Photoshop CS5 software (Adobe Systems, San Jose, CA, USA).

## Abbreviations

CLSM: Confocal laser scanning microscopy; HAT: Histone acetyltransferase; HDAC: Histone deacetylase; IES: Internal eliminated sequences; MDS: Macronuclear-destined sequence; PCR: Polymerase chain reaction; PTM: Posttranslational modification; RP-HPLC: Reverse phase-high performance liquid chromatography; TSA: Trichostatin A

## Competing interests

The authors declare that they have no competing interests.

## Authors’ contributions

AB, JP, AF and FJ performed and designed experiments. GR and HJL made the overall design and coordination of the study. HJL, in collaboration with all authors, wrote the manuscript which was approved by them all.

## Supplementary Material

Additional file 1 **5meC quantification in *mdp1 *and *mpd2*.** Click here for file

Additional file 2 **DNA methylation patterns of A. *mdp1*, B. *mdp2 *and C. *alpha-tubulin *in differentiating macronuclei (anlagen).** Click here for file

Additional file 3 **Effect of C646 on the distribution of H3K9ac/K14ac on A. *mdp1 *and B. *mdp2*.** Click here for file
