# Supplementary material for: A permissive chromatin structure is adopted prior to site-specific DNA demethylation of developmentally expressed genes involved in macronuclear differentiation
Source: Epigenetics Chromatin. 2013 Mar 5;6:5. doi: 10.1186/1756-8935-6-5 (PMC3608066; doi:10.1186/1756-8935-6-5)
Supplement: Additional file 1 — 5meC quantification in mdp1 and mpd2. [file 1756-8935-6-5-S1.pdf]

Additional files

Additional file 1 – 5meC quantification in *mdp1* and *mpd2*

mdp1 nanochromosome

Vegetative

- 18 sequences total, all sequences contain methylated cytosines.
- 73% of all 5'-cytosines are methylated.
- 5x 1 cytosine methylated=28%; 9x 2 cytosines methylated=50%; 4x 3 cytosines methylated=22%.
- 1st position cytosine methylated=56%; 2nd position cytosine methylated=72%; 3rd position cytosine methylated=67% .

Exconjugant

- 18 sequences total, 78% contain no methylated cytosine.
- 4 sequences (22%) contain a single methylated cytosine at 1st or second position.

Exjonjugant HAT inhibitor C646

- 16 sequences total, all sequences contain methylated cytosines.
- 46% of all 5'-cytosines methylated.
- 12x 1 cytosine methylated=75%; 2x 2 cytosines methylated=13%; 2x 3 cytosines methylated=13%.
- 1st position cytosine methylated=56%; 2nd position cytosine methylated=19%; 3rd position cytosine methylated=63% .

Exconjugant HDAC inhibitor TSA

- 8 seqs; no methylated cytosines found.

mdp2 **nanochromosome**

Vegetative

- 19 sequences total, 63% of all sequences contain a single methylated cytosine, whereas no cytosine methylation could be detected in the remaining sequences.

Exconjugant

- 15 sequences total, no cytosine methylation could be detected in 93% of all sequences.

Exjonjugant HAT inhibitor C646

- 8 sequences total, 88% of all sequences contain a single methylated cytosine.

Exconjugant HDAC inhibitor TSA

- 8 seqs; no methylated cytosines found.
