# Supplementary material for: A permissive chromatin structure is adopted prior to site-specific DNA demethylation of developmentally expressed genes involved in macronuclear differentiation
Source: Epigenetics Chromatin. 2013 Mar 5;6:5. doi: 10.1186/1756-8935-6-5 (PMC3608066; doi:10.1186/1756-8935-6-5)

Additional file 2 – DNA methylation patterns of A. *mdp1*, B. *mdp2* and C. *alpha-tubulin* in differentiating macronuclei (anlagen). In each cartoon the first line is the bisulfite-untreated original sequence, which in these samples was not excluded from the consensus sequence illustration (on top).

A.

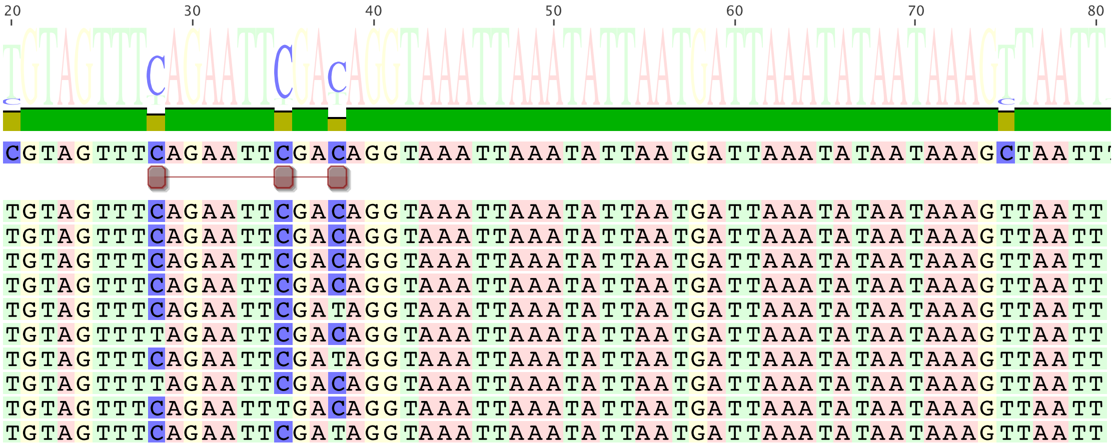

B.

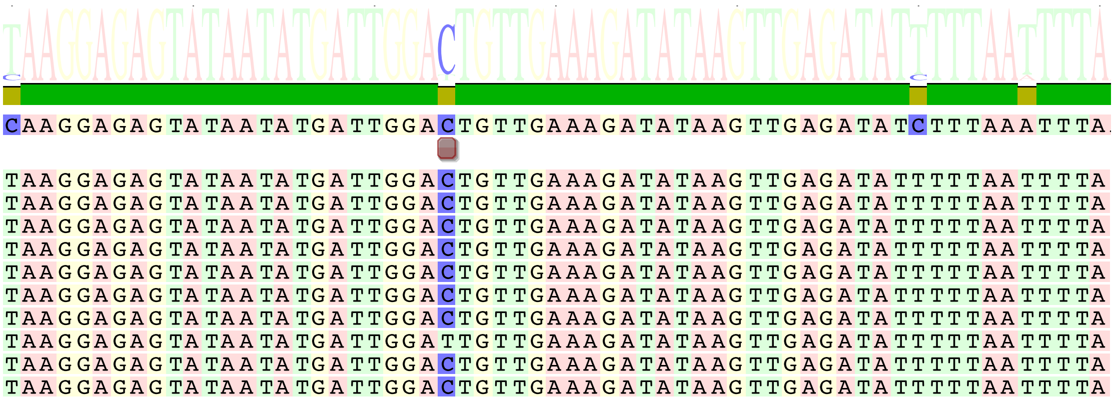

C.

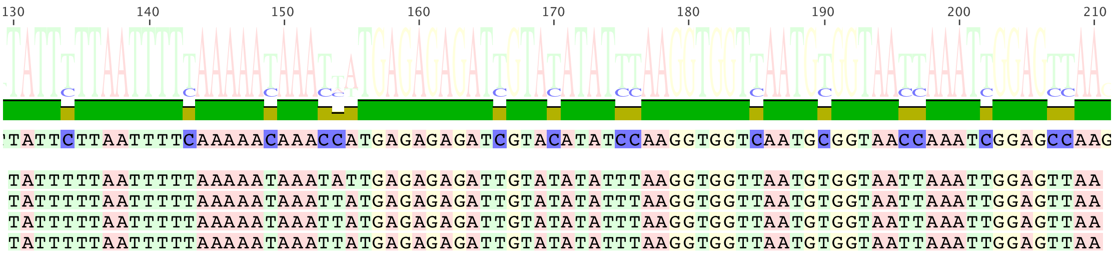

Supplement: Additional file 2 — DNA methylation patterns of A. mdp1, B. mdp2 and C. alpha-tubulin in differentiating macronuclei (anlagen). [file 1756-8935-6-5-S2.pdf]
