# Supplementary figures and images for: A permissive chromatin structure is adopted prior to site-specific DNA demethylation of developmentally expressed genes involved in macronuclear differentiation
Source: Epigenetics Chromatin. 2013 Mar 5;6:5. doi: 10.1186/1756-8935-6-5 (PMC3608066; doi:10.1186/1756-8935-6-5)

**Additional file 3 – Effect of C646 on the distribution of H3K9ac/K14ac on A. *mdp1* and B. *mdp2***

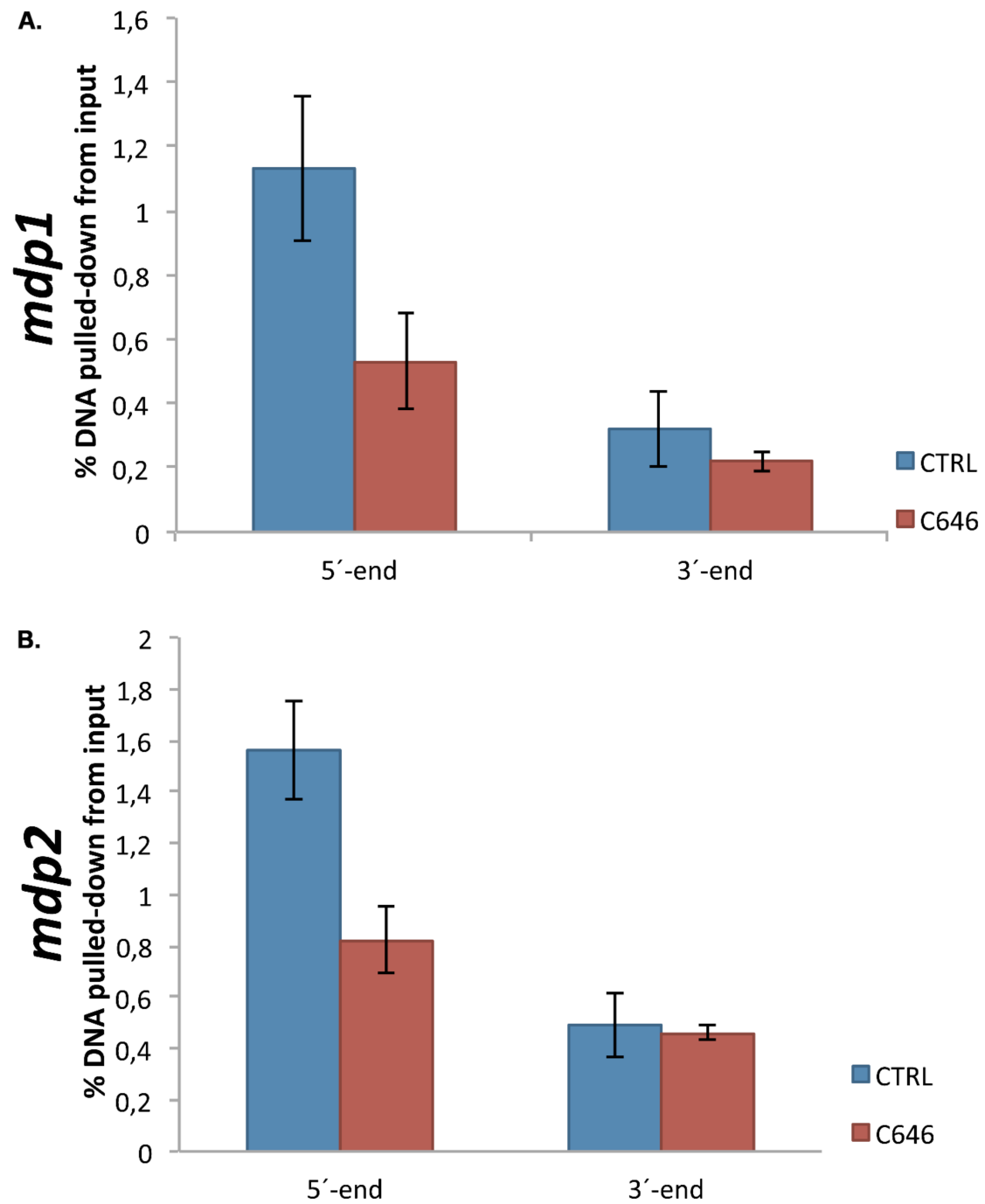

Supplement: Additional file 3 — Effect of C646 on the distribution of H3K9ac/K14ac on A. mdp1 and B. mdp2. [file 1756-8935-6-5-S3.pdf]
